# Closed-Loop Valorization of Urban Sewage Sludge for Sustainable Biodiesel Production

**DOI:** 10.3390/molecules31183273

**Published:** 2026-09-15

**Authors:** Carlo Pastore, Luigi di Bitonto, Valeria D’Ambrosio, Antonella Angelini, Felipe de Jesús Villalobos-Delgado, Didilia Ileana Mendoza-Castillo, Hilda Elizabeth Reynel-Ávila, Maria Lacalamita, Jakub Vagunda, Martin Hájek, Adrián Bonilla-Petriciolet

**Affiliations:** 1 Water Research Institute (IRSA), National Research Council (CNR), Via F. de Blasio 5, 70132 Bari, Italy; luigi.dibitonto@cnr.it (L.d.B.); valeriadambrosio@cnr.it (V.D.); antonella.angelini@cnr.it (A.A.); 2 Department of Chemical Engineering, Instituto Tecnológico de Aguascalientes, Aguascalientes 20256, Mexico; iqfelipedejesusvillalobosiq@hotmail.com (F.d.J.V.-D.); didi_men@hotmail.com (D.I.M.-C.); elizabethreynel@gmail.com (H.E.R.-Á.); petriciolet@hotmail.com (A.B.-P.); 3 Secretary of Science, Humanities, Technology and Innovation (SECIHTI), Mexico City 03940, Mexico; 4 Department of Earth and Geoenvironmental Sciences, University of Study of Bari “Aldo Moro”, Via Orabona 4, 70125 Bari, Italy; maria.lacalamita@uniba.it; 5 Faculty of Chemical Technology, University of Pardubice, Studentska’ 573, 532 10 Pardubice, Czech Republic; jakub.vagunda@upce.cz (J.V.); martin.hajek@upce.cz (M.H.)

**Keywords:** sewage sludge, solvent-free lipid recovery, biodiesel, zero waste discharge, circular economy, biochar, pyrolysis, sulfonation

## Abstract

The environmental and economic challenges stemming from fossil fuel dependence and climate change highlight the urgent need for sustainable biofuels. Wastewater treatment byproducts, such as sewage sludge (SS), represent a promising, yet underutilized, feedstock for biodiesel production within a circular economy. This research proposes a closed-loop valorization strategy for wastewater treatment byproducts, integrating solventless lipid extraction with the pyrolysis (at 400, 550, and 750 °C) of lipid-free residual biomass to produce the relevant biochars (BC). BCs can be functionalized (sulfonation run at 175 °C, 24 h), characterized (CHNS, FTIR, XRD, SBET, TPD, SEM), and used as catalysts for the esterification reaction of the previously extracted lipids with methanol to produce biodiesel. The extracted lipids were reacted with methanol (1:10 molar ratio, 80 °C, 5 h) using 3 wt.% of sulfonated catalysts per gram of grease, producing fatty acid methyl esters in high yield and conversion (>96%). Catalysts were easily and efficiently recoverable through centrifugation, rendering them stable and reusable for five cycles without losing their catalytic properties. This approach aims to transform SS into a valuable resource, fostering a more sustainable and efficient biofuel production system.

## 1. Introduction

The growing environmental concerns associated with fossil fuel dependency and climate change necessitate the development of sustainable biofuels. Fossil fuels, including natural gas, coal, and crude oil, account for more than 80% of the total global energy production [1]. It is imperative to reverse this trend by increasing investments in biofuel production while identifying feedstocks that are not only sustainable and renewable but also economically competitive with fossil fuels [2]. Waste biomass could meet these criteria, given its widespread availability, zero cost, and the economic advantage derived from waste volume reduction when repurposed as raw material [3]. Among these, wastewater treatment by-products emerge as innovative alternatives. Wastewater treatment plants (WWTPs) generate significant amounts of primary sewage sludge, representing the major wastewater treatment by-product in which most of the solids from raw sewage accumulate [4]. This substrate is also rich in lipidic compounds (20–50 wt.% relative to dry matter [5]), making it a highly promising feedstock for biodiesel production. Given the urgent need for sustainable energy solutions, the exploitation of these waste-derived lipid resources could represent a crucial step toward a circular and economically viable biofuel industry. However, the challenge in utilizing these by-products for biofuel production lies in optimizing extraction and conversion techniques to ensure a sustainable and cost-effective valorization process [6].

Studies have been conducted on the recovery of desirable compounds using various processing routes. A promising solvent-free methodology for lipid extraction was recently reported [7,8] and could be applied to sewage scum and primary sludge. This process involves the acidification (with HCl) and oxidation (H_2_O_2_) of these by-products, followed by heating at 80 °C and subsequent centrifugation, leading to the recovery of lipids as the upper phase of a three-phase system [7,8,9]. The extracted lipid fraction, with a purity of over 90% in fatty acids, represents a suitable feedstock for biodiesel production. Beyond lipid extraction, the residual lipid-free biomass (approximately 35% of the original wastewater-derived by-product) requires further treatment [8]. To better integrate the solvent-free process within the WWTP framework and move toward the ambitious goal of zero-waste discharge, this residual biomass should be further valorized. Besides its integration into conventional anaerobic digestion, studies have shown that, after drying, the degreased residual sludge can be used as a secondary solid fuel capable of meeting the energy demand of the entire sludge treatment process. However, this approach is limited to energy recovery.

To maximize the value of the residual carbon retained in the biomass, pyrolysis represents a promising alternative [10]. This thermochemical conversion process enables carbon sequestration through the production of biochar while simultaneously generating pyrolysis oil (bio-oil) and pyro-gas (syngas), both of which can be recovered as valuable energy carriers or feedstock for producing new chemicals. Such a strategy may offer the best compromise between material and energy recovery, while also ensuring the effective degradation of persistent organic pollutants (POPs) present in sewage sludge, an issue that has received increasing attention in recent years.

In addition, one potential approach for preparing novel catalysts involves pyrolysis of the residual biomass to produce biochar, which, upon functionalization with suitable functional groups, could act as an effective esterification catalyst, as already demonstrated in several studies [11]. Particularly, sulfonated biochar has emerged as the most widely used heterogeneous catalyst for esterification reactions. The introduction of –SO_3_H groups onto the biochar surface during the sulfonation process, typically via H_2_SO_4_ treatment or exposure to gaseous SO_3_ [12,13,14], significantly enhances its catalytic activity, demonstrating remarkable conversion of fatty acids to methyl esters (90–100%), even under mild conditions (55–60 °C) [12]. Furthermore, the presence of metals and other inorganic compounds in the biochar derived from residual sludge could further enhance its catalytic performance in esterification and transesterification reactions. Recently, sewage sludge has been investigated as a catalyst for the (trans)esterification of lipids after being degreased by solvent extraction and pyrolyzed at 600 °C [15]. The resulting biochar promoted the (trans)esterification of oils due to its intrinsic physical properties, such as porosity and surface area, as well as its chemical characteristics, particularly the presence of basic active sites.

In the present work, for the first time, an innovative integrated process was designed and experimentally evaluated. The proposed approach involves: (i) the recovery of grease from wet sewage sludge through a solvent-free procedure, and (ii) the subsequent pyrolysis and sulfonation of the exhausted residual sludge to produce a catalyst for the direct esterification under mild reaction conditions, enabling the sequential valorization of both the lipid and solid fractions of sewage sludge within a single process. In conclusion, the valorization of wastewater treatment by-products, particularly sewage scum and primary sewage sludge, presents a promising strategy for sustainable biofuel production. By extracting lipids and exploring innovative approaches such as utilizing lipid-free residual biomass for catalytic purposes, these waste materials can be fully transformed into valuable resources. This not only addresses the environmental concerns these wastes typically imply but also contributes to the development of a circular biofuel economy, enhancing both economic and ecological sustainability. This approach enabled a broad assessment of the valorization and utilization of these residues as feedstocks for the development of catalytic materials.

## 2. Results

### 2.1. Solvent-Free Recovery of Grease from Urban Sewage Sludge and Chemical Characterization of Degreased Sewage Sludge

Grease recovery from urban sewage sludge was carried out at pilot scale (2 t h^−1^) using a solvent-free process. Sedimented sewage sludge (total solids, TS: 2.2 wt.%; lipid content: 25.1 wt.%) was first dewatered by centrifugation, yielding a centrifuged sludge with a TS content of 19.8 wt.% and a grease content of 25.0 wt.%, and a centrate (Centrate 1) which could be sent back to the head of the WWTP. After approximately 700 kg of dewatered sludge had accumulated, the sludge was acidified with hydrochloric acid (33 wt.%, 50 kg) and treated with hydrogen peroxide (35 wt.%, 25 kg). The mixture was then heated to 80 °C and centrifuged, producing three distinct streams: (i) raw grease (38.6 kg; free fatty acid, FFA, content: 92.3 wt.%), (ii) an exhausted aqueous phase (560 kg), and (iii) a dewatered degreased residual sludge (217 kg; TS: 32 wt.%). Based on these mass balances, the grease recovery yield reached 72 wt.%. The resulting dewatered sludge was subsequently dried using a low-temperature SHINCCI Model SBDD2400FL pilot-scale drying unit. This system employs forced recirculation of hot air to directly heat the sludge, providing high thermal efficiency while operating at relatively low temperatures. Drying was performed at 75 °C. Approximately 74 kg of dried sludge, with a residual moisture content of 6.2 wt.%, was recovered, while consuming about 50 kWh [8]. The dried material was then chemically characterized, ground, and used in the subsequent experimental activities. Table 1 reports the details of the characterization analysis determined on the dried degreased sludge recovered after in situ desiccation.

Table 1 shows that in the residual degreased sludge, a list of compounds remained present: total lipids, easily hydrolysable sugars, cellulose, proteins, and ligno-humic compounds prevalently constituted such a residue. As for the mineral component, the elemental composition analysis of lipid-free residues reported in Table 2 reveals a significant concentration of mineral elements with potential catalytic relevance for biodiesel production. Calcium emerged as the most abundant element, with concentrations exceeding 17,000 mg/kg in the lipid-free residue. Iron also showed considerable presence, with concentrations around 5758 mg/kg. Iron and calcium can contribute to catalytic activity by forming mixed metal oxide species.

It is expected that these residues could be a promising precursor in the synthesis of metal oxide-based catalysts. The elevated aluminum content suggests the formation of complex mineral phases [16]. Studies conducted on various samples of sewage sludge have confirmed the presence of these three inorganic elements in high abundance [17]. Elements Ag, As, Be, Co, Sb, Se, Cd, Ti, and Tl were reported at levels below 0.1 mg/kg.

### 2.2. Pyrolysis of Residual Degreased Sludge and Preliminary Characterization

Pyrolysis of the residual degreased sludge was operated at 400, 550, and 750 °C, producing carbonaceous materials (pyro-chars) with solid yields of 31–49 wt.%. A decrease in yield was observed with increasing temperature. This is consistent with the fact that lower temperatures favor pyro-char generation, while higher temperatures favor gas formation from the pyrolyzed biomass [18]. Table 3 collects the determined yields of the pyro-chars, pyro-oils, and the relevant gaseous phases at 400, 550, and 750 °C.

**Figure 1:**
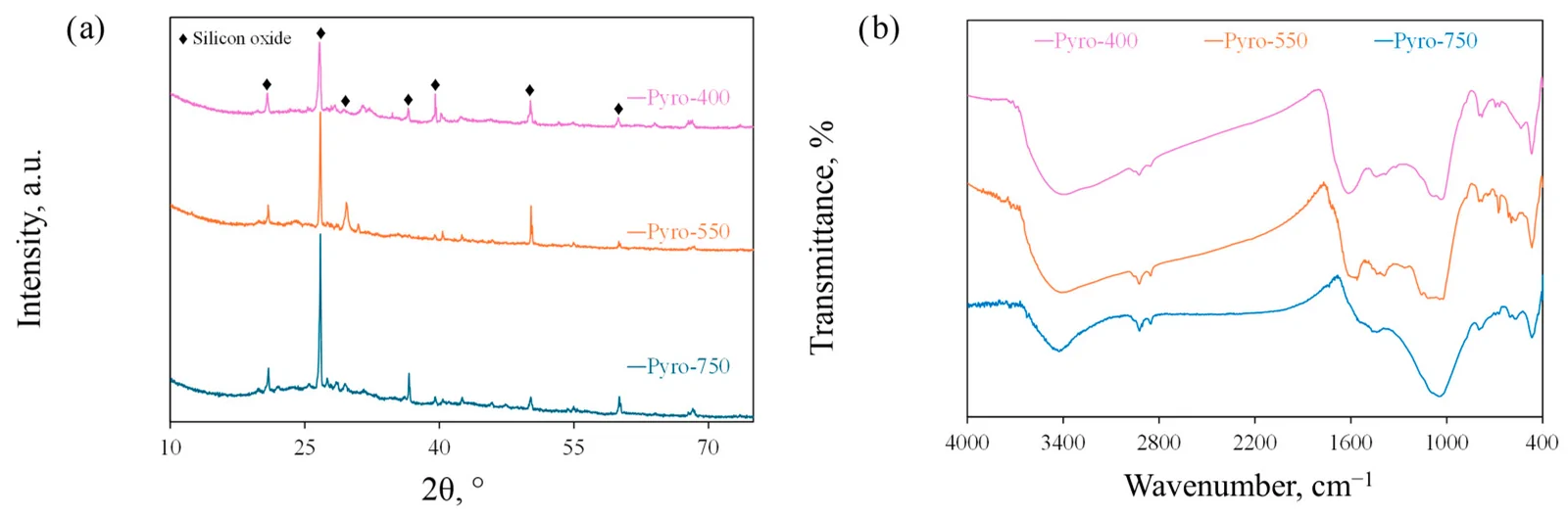
(**a**) X-ray diffraction patterns and (**b**) FTIR spectra of pyro-chars derived from the degreased residues at 400, 550, and 750 °C.

**Figure A1:**
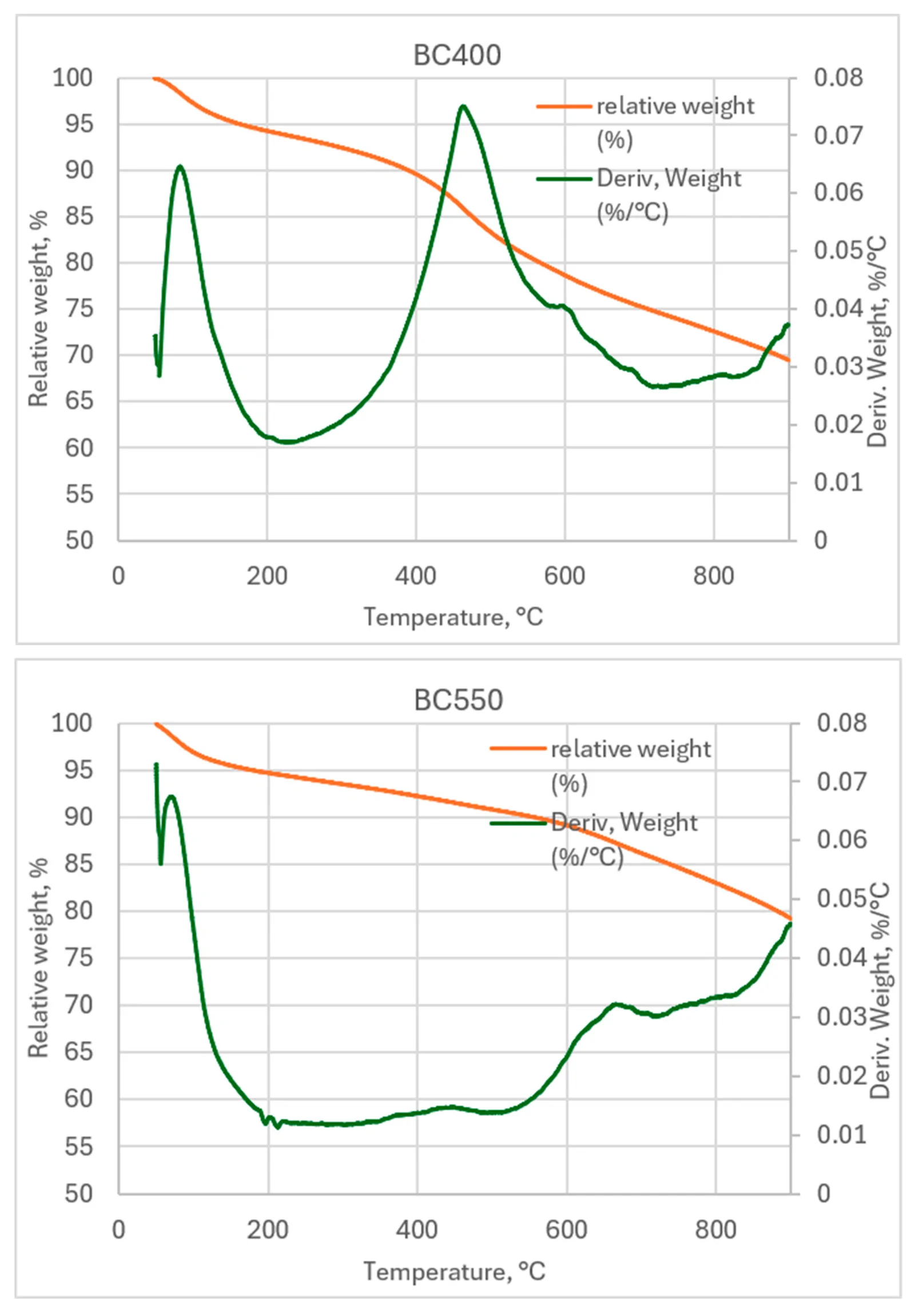
TGA profiles for BC400 and BC550.

As reported in Table 3, a significant yield of pyrolysis oil (bio-oil) was obtained only when the process was carried out at 400 °C. This liquid fraction consisted predominantly of residual free fatty acids (FFAs), which accounted for more than 65 wt.% of the bio-oil. Overall, approximately 52% of the FFAs initially present in the solvent-free degreased sludge were recovered in this fraction. As the pyrolysis temperature increased, the yield of pyro-char progressively approached the ash content of the feedstock, indicating that an increasing proportion of the organic matter and its associated energy was converted into the gaseous phase. Thermogravimetric analysis (TGA) showed that more than 60 wt.% of the pyro-char produced at 750 °C consisted of ash and other inorganic mineral components. On the contrary, BC400 and BC550 contained more degradable components, namely 35 and 20%, respectively (Figure A1). In detail, BC400 contains components that are thermally degradable at 500 °C, which are naturally absent in BC550. These components may provide additional organic sites onto which sulfonic groups can be grafted during the sulfonation process. These findings were further supported by X-ray diffraction (XRD) and Fourier-transform infrared (FTIR) spectroscopy analyses, which confirmed the predominance of the mineral fraction in the high-temperature pyro-chars. Figure 1 shows the X-ray diffractograms of the carbon-based materials synthesized from sewage sludge by pyrolysis at different temperatures.

All pyrochars exhibited diffraction peaks at ~26.7, ~36.6, ~39.5, ~42.5, ~50.1, and ~54.9 °2θ, with hkl values (011), (110), (102), (200), (112), and (022) attributed to silicon oxide (ICDD: 01-085-0794) with hexagonal structure. These samples also evidence the coexistence of an amorphous matrix and different crystalline mineral phases. In particular, the broad diffraction peak located between ~18 and ~30 °2θ may be indexed to carbon structures related to the (002) plane [19]. This is related to disordered organic carbon, as well as residual organic content. Similar diffraction behavior has been observed in carbon-based materials derived from sewage sludge, where thermal carbonization promotes the development of partially ordered aromatic domains without achieving a highly graphitic structure [20,21].

Furthermore, an increase in crystallinity was observed with the rise in the pyrolysis temperature due to the progressive thermal decomposition of volatile organic components, the simultaneous carbonization of organic matter, and the relative enrichment and crystallization of minerals within the solid matrix [22].

Figure 1b shows the FTIR spectra of the synthesized materials. All catalysts exhibit the absorption band associated with the stretching vibrations of O-H (~3440 cm^−1^) from hydroxyl groups, adsorbed water, phenolic groups, and possibly N-H bonds from protein-derived residues [20,23,24].

The spectra of these materials also display absorption bands associated with asymmetric and symmetric C-H (~2925–2850 cm^−1^) stretching vibration of aliphatic -CH_2_ and CH_3_ groups, C=O stretching of carboxyl and carbonyl groups together with C=C vibrations of aromatic structures (~1700–1500 cm^−1^), Si-O-Si (~1100–1000 cm^−1^) and Si-O (~790 cm^−1^) bonds, and metal-oxygen (~750–400 cm^−1^) vibrations [20,23,24]. The metal-oxygen bonding results from the inorganic matter (i.e., Al, Ca, Na) of the feedstock due to the mineral-rich nature of sewage sludge. These results are consistent with those reported by Campos-Avanzi et al. [25] and Vali et al. [26].

In general, with the increase in the temperature of thermal treatment, the intensity of hydroxyl, amine, and aliphatic functional groups decreased due to the decomposition of the organic matter present in the sludge matrix. The gradual disappearance of these surface functionalities indicates the progression of dehydration, decarboxylation, deamination, and aromatization reactions during the thermochemical conversion process [27]. Similar trends have been reported for different sewage sludge-derived biochars [20,22,23,26]. The opposite behavior was observed for the mineral phase. This behavior can be attributed to the enrichment of the ash-forming inorganic fraction of the precursor [25]. Consequently, the mineral phase becomes more concentrated within the solid matrix, increasing the contribution of inorganic vibrations. For these reasons, the characterization and functionalization were mainly focused on Biochar obtained at 400 and 550 °C, namely BC400 and BC550, respectively, leaving out BC750.

### 2.3. Functionalization of Pyrolyzed Residual Degreases Sludge and Chemical Characterization

BC400 and BC550 were treated with a large excess of H_2_SO_4_ (conc.) (1:50 m:v) at 175 °C for 24 h, obtaining black fine powders marked with BC400S and BC550S, respectively, with an overall yield of about 50%. Such a loss of weight was mainly due to the consistent solubilization of the initial organic and inorganic soluble components. Table 4 reports the CHNS analysis of BC400, BC550, and the relevant sulphonated compounds.

**Figure A2:**
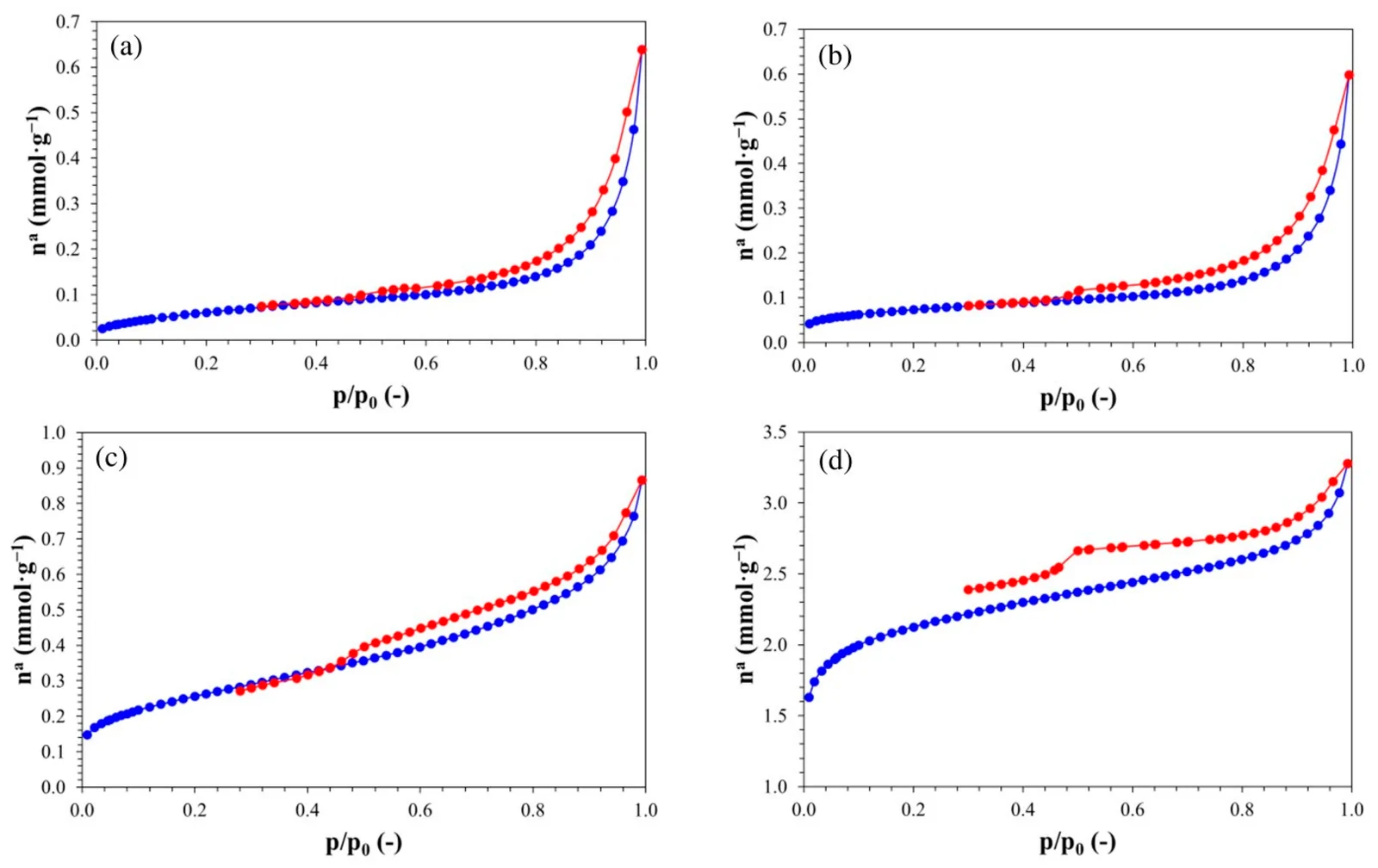
Linear plot of isotherms related to (**a**) BC400, (**b**) BC550, (**c**) BC400S, and (**d**) BC550S. The blue and red curves correspond to the N_2_ adsorption and desorption branches, respectively.

More specifically, as reported in Table 4, the sulfonation of BC400 did not significantly affect its initial carbon content, whereas BC550S exhibited a more pronounced increase in carbon concentration as a result of a preferential dissolution of the mineral fraction during the sulfonation treatment. Nevertheless, sulfonation was more effective for BC400, as evidenced by its sulfur content, which was approximately 30% higher than that measured for BC550S. In agreement with these figures, nitrogen physisorption revealed a significant effect of sulfonation on the textural properties of the prepared pyro-chars. The original BC550 and BC400 exhibited very low specific surface areas (5.9 and 5.2 m^2^·g^−1^), suggesting a predominance of inaccessible or macroporous structures. After sulfonation, the specific surface area increased for both samples; for the BC550S sample, the increase was exceptionally pronounced, reaching 178.0 m^2^·g^−1^, while for BC400S it reached only 20.5 m^2^·g^−1^. These results suggest that sulfonation not only led to the introduction of sulfonic groups onto the material’s surface but also likely removed some of the deposits or mineral components, thereby opening up previously closed pores. The markedly different responses of the BC550 and BC400 samples also indicate that the effectiveness of sulfonation is strongly dependent on the initial structure of the biochar. The adsorption and desorption isotherms for nitrogen in the BC550S sample exhibit a characteristic Type IV(a) hysteresis loop, which confirms the presence of a mesoporous structure supplemented by a significant proportion of micropores (Figure A2). An interesting feature is that the adsorption and desorption curves do not overlap in the region of relative pressures approximately p/p0 = 0.3–0.5, and the desorption curve remains above the adsorption curve. At the same time, a relatively sharp drop in the desorption curve is evident around p/p0 ≈ 0.5, which can be attributed to the network effect and pore blocking in a complex network of interconnected mesopores with narrow necks (so-called ink-bottle pores). In this region, cavitation (tensile strength effect) of liquid nitrogen may also occur, causing the sudden emptying of some of the pores. The behavior described is common in heterogeneous carbonaceous materials with a hierarchical micro-mesoporous structure and does not in itself indicate a measurement error. On the contrary, it suggests that sulfonation significantly altered the character of the BC550 sample’s pore structure and led to the formation of a complex network of accessible pores.

**Figure A3:**
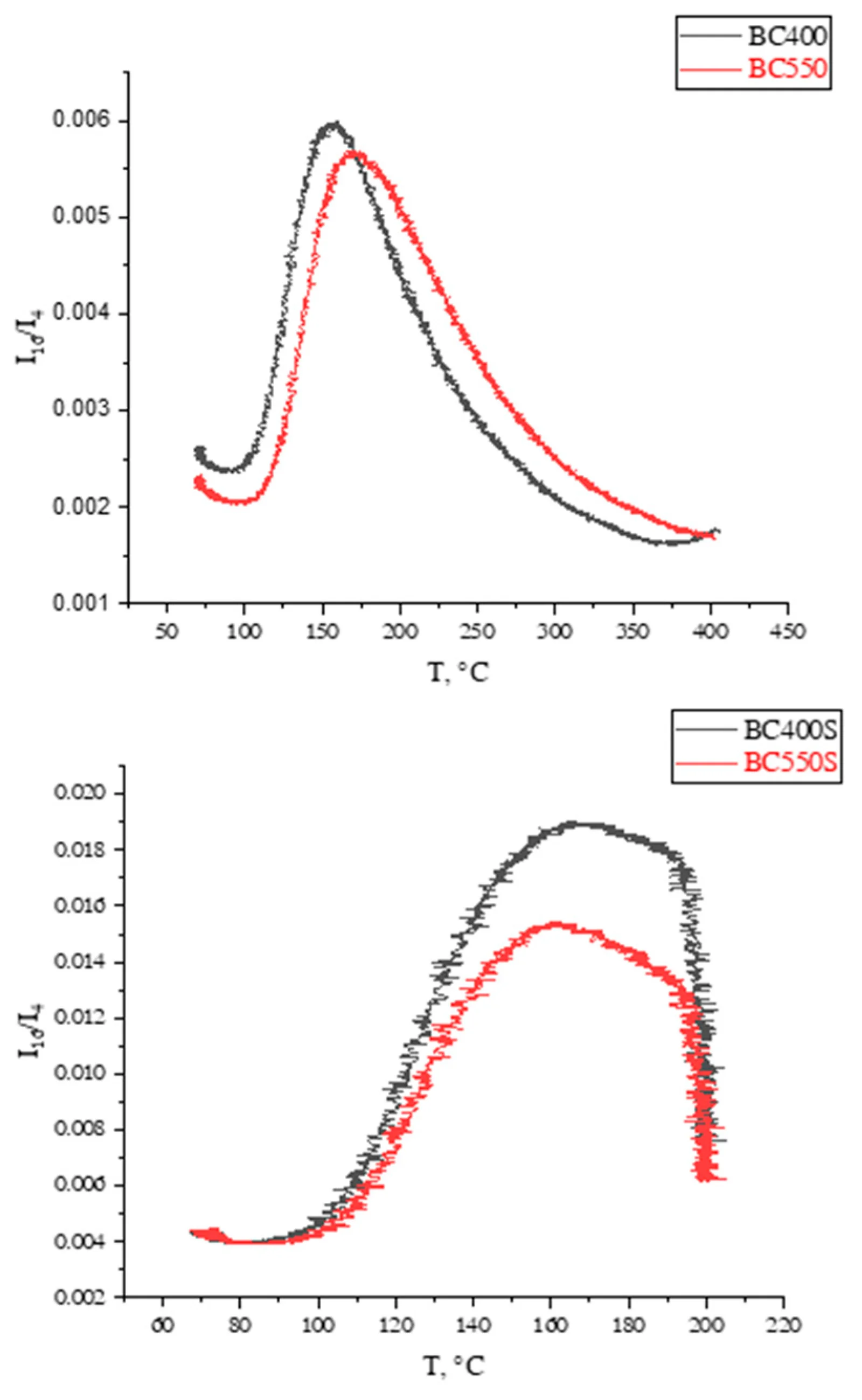
TPD-NH_3_ profiles of BC and BCS samples.

For the BCS samples, pretreatment was performed at 170 °C. Due to the tailing of the desorption peak, the concentration of acidic sites (CAS) was evaluated over a temperature range of 25–200 °C to capture the entire peak area (Figure A3). However, it should be noted that at higher temperatures, the signal may also be partially influenced by the desorption of other volatile components from the sample that were not removed by pretreatment above 170 °C, which may lead to a slight overestimation of the calculated acid center concentration. Furthermore, there is also the effect of matrix decomposition (sulfonate groups).

**Figure A4:**
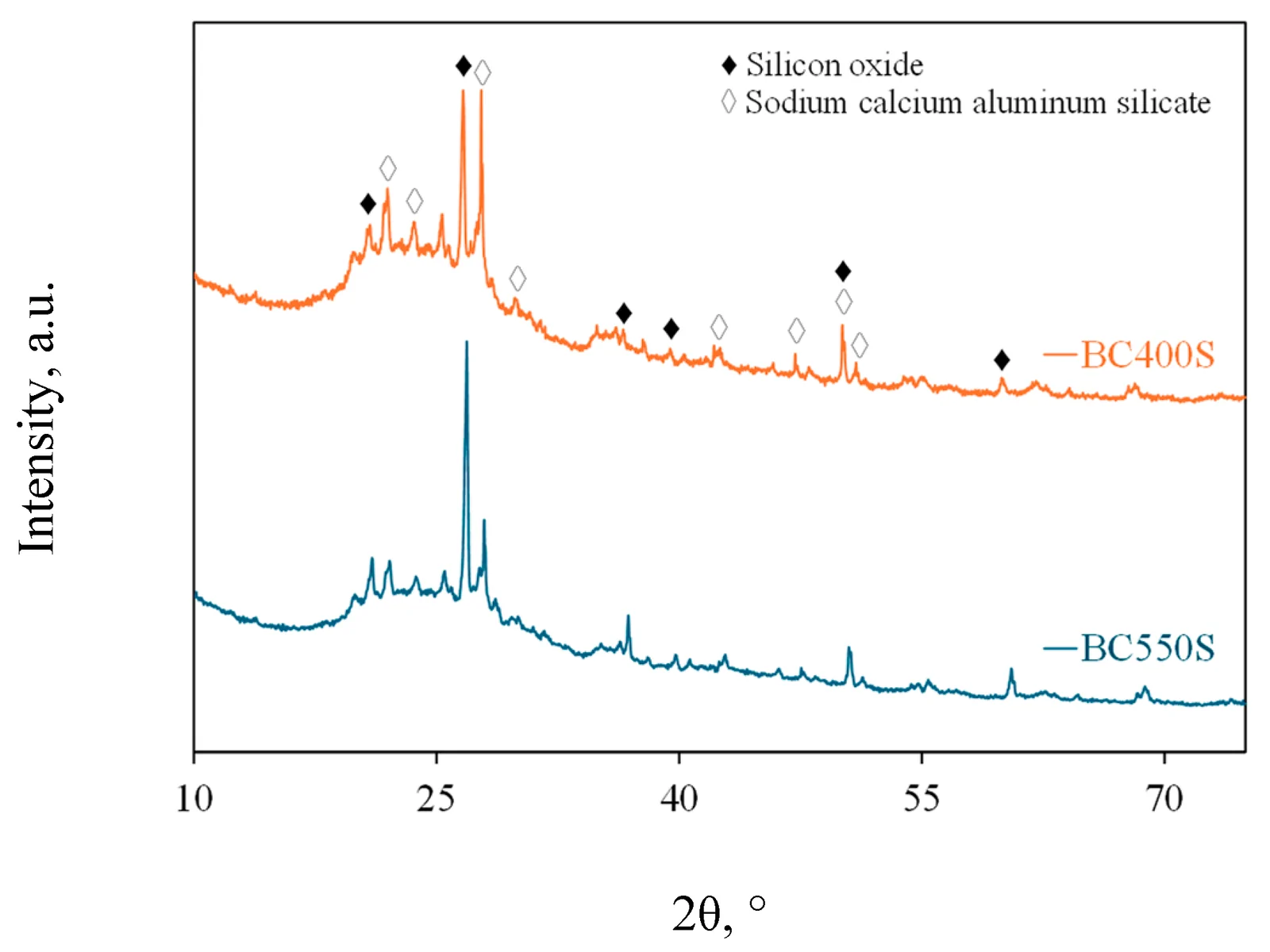
XRD profiles of BCS samples.

XRD analysis show some changes in the crystalline structure and/or crystallinity were identified after surface modification with sulfuric acid. According to the literature, this acid can induce mineral dissolution, leading to variations in peak intensity and diffraction profile [28] (Figure A4).

**Figure 2:**
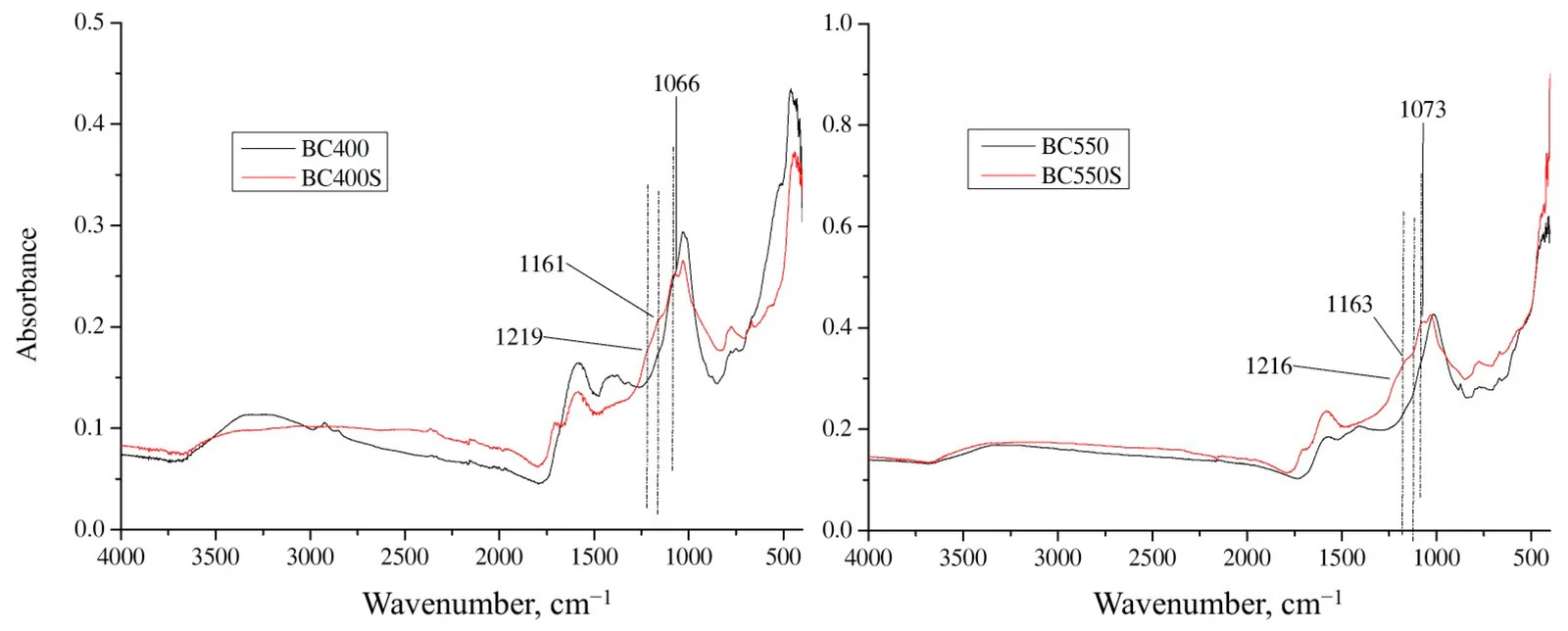
FTIR spectra comparison between BC400, BC550 and the relevant sulphonated forms.

**Figure A5:**
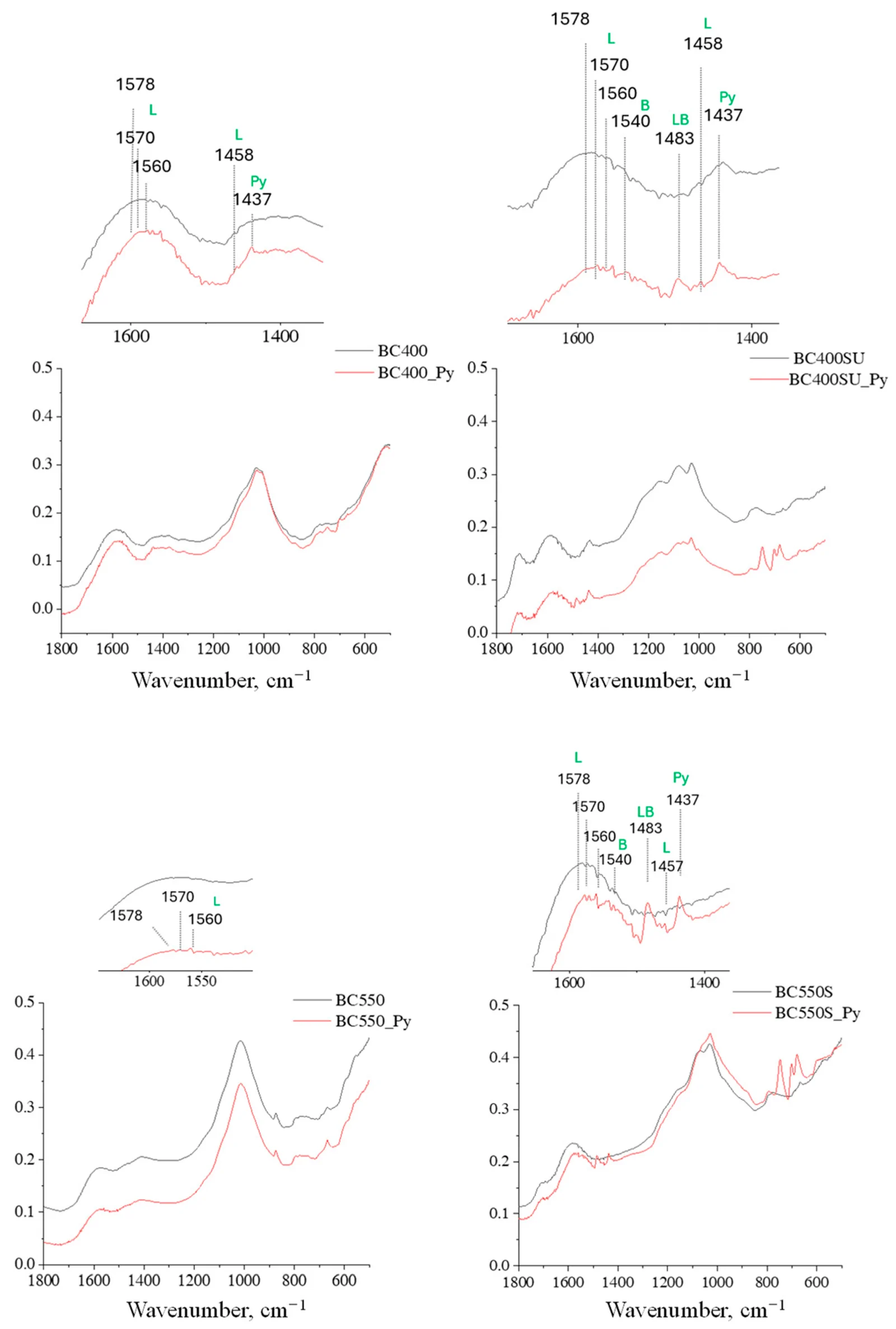
FTIR profiles of BC, BCS, and BCSU samples with pyridine adsorption (L: Lewis sites; B: Brønsted sites; LB: Lewis-Brønsted sites; Py: Physiosorbed Pyridine).

The FTIR spectra of raw and sulfonated biochars are shown in Figure 2. As regards the sulfonated species, bands at 3418 and 1610 cm^−1^, attributable to the stretching and bending vibrations of the -OH, -NH, and -COOH groups, respectively, still remain after sulfonation. However, after the sulfonation process, the FTIR spectra of the sulfonated biochars exhibited characteristic absorption bands at 1219, 1161, and 1066 cm^−1^, which are assigned to the asymmetric and symmetric stretching vibrations of the O=S=O group and the S–O stretching vibration of sulfonic acid (–SO_3_H) functionalities. The appearance and increased intensity of these bands after sulfonation confirm the successful grafting of sulfur-containing acidic groups onto the biochar surface [29,30]. FTIR was also used to better define the nature of the acid sites, using pyridine as a probe molecule (Figure A5). The solid samples were exposed to pyridine vapor at room temperature for 30 min, followed by desorption under a nitrogen flow at 100 °C to remove weakly physiosorbed pyridine [31]. The IR spectrum of the precursor showed only very weak absorption bands in the 1560–1580 cm^−1^ region, indicating the presence of a limited amount of acid sites. After the sulfonation, the FTIR spectra exhibited the appearance of new characteristic bands at 1540 cm^−1^, assigned to pyridinium ions formed on Brønsted acid sites; at 1457 cm^−1^, attributed to pyridine coordinated to Lewis acid sites; and at 1483 cm^−1^, associated with pyridine interacting with both Brønsted and Lewis acid sites [31,32,33,34]. Among these, the band at 1483 cm^−1^ was the most intense, suggesting that sulfonation increased the overall surface acidity, with the coexistence of both Brønsted and Lewis acid sites. The used sulfonated biochar retained the same spectral profile after catalytic use.

**Figure 3:**
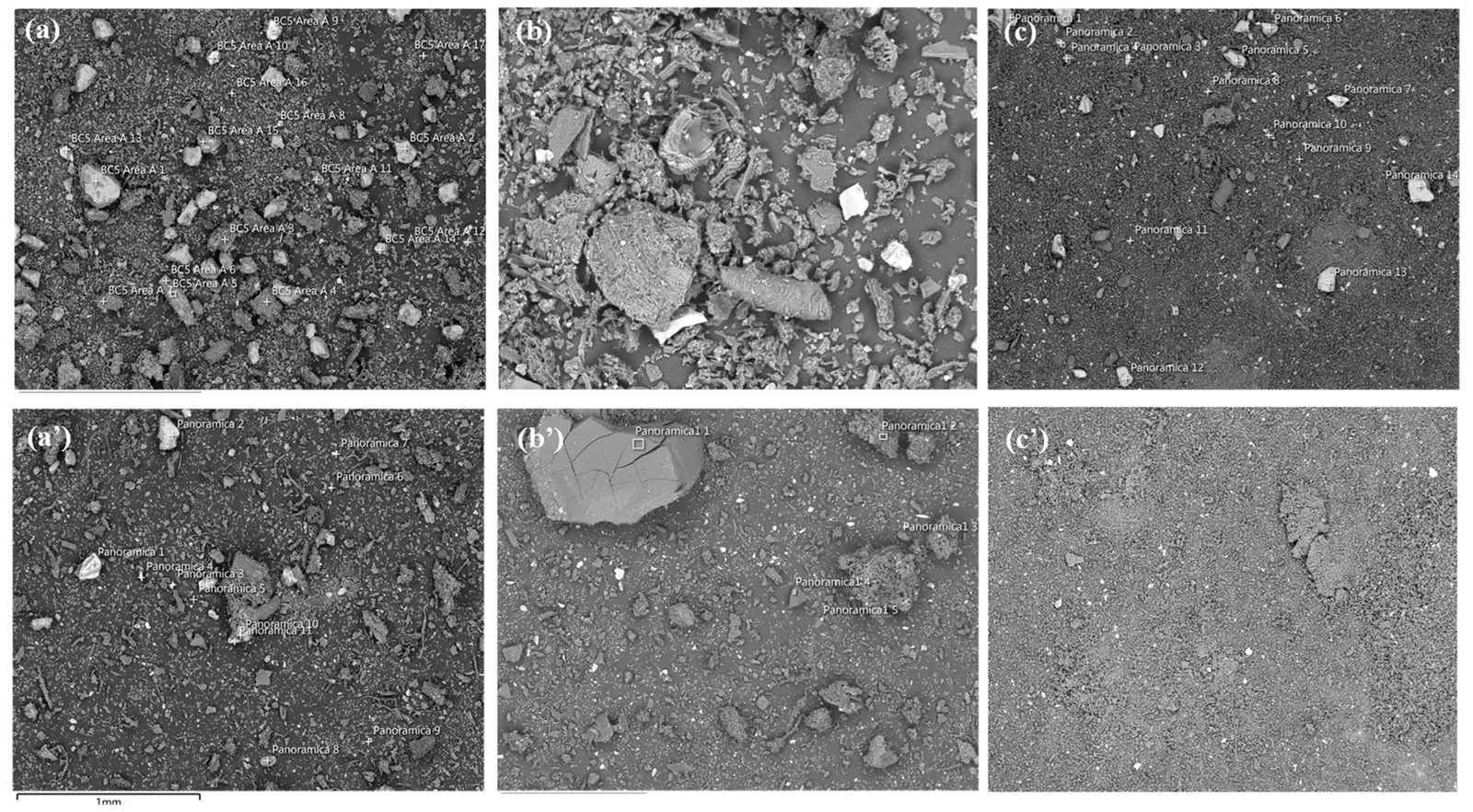
SEM images on BC550 (**a**), B550S (**b**), BC550SU (**c**), BC400 (**a’**), BC400S (**b’**), BC400SU (**c’**).

Figure 3 shows the morphology of the initial biochars (BC), the relevant sulfonated systems (BCS), and the sulfonated samples recovered after reuse in catalysis (BCSU). The micrographs reveal a carbonaceous biochar matrix with several mineral phases dispersed throughout its structure. In particular, the brighter regions are attributable to silica-rich mineral particles, owing to their higher electron density.

**Figure A6:**
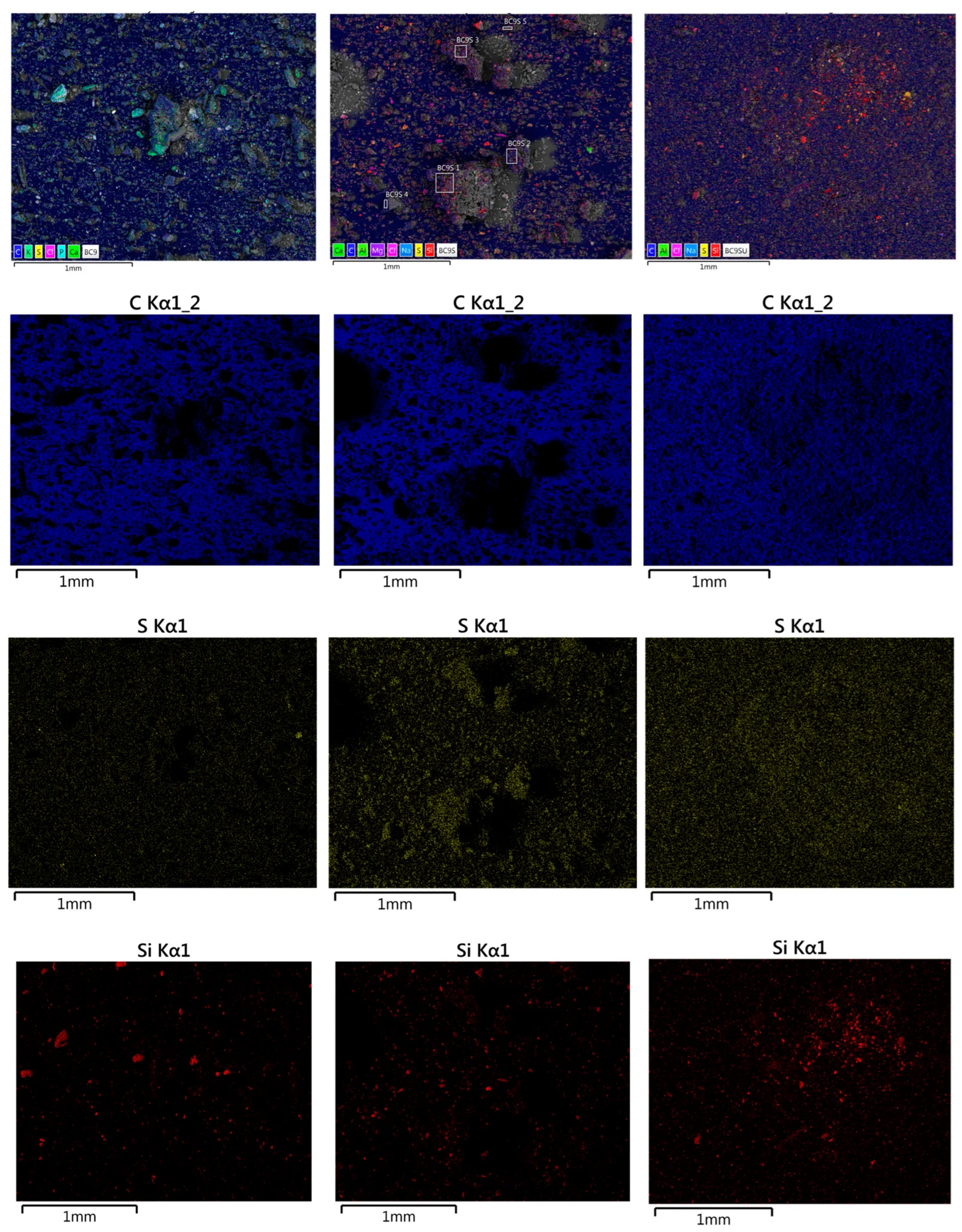
SEM images of BC400, BC400S, and BC400SU; mappings of C, S, and Si were reported in the correspondence.

**Figure A7:**
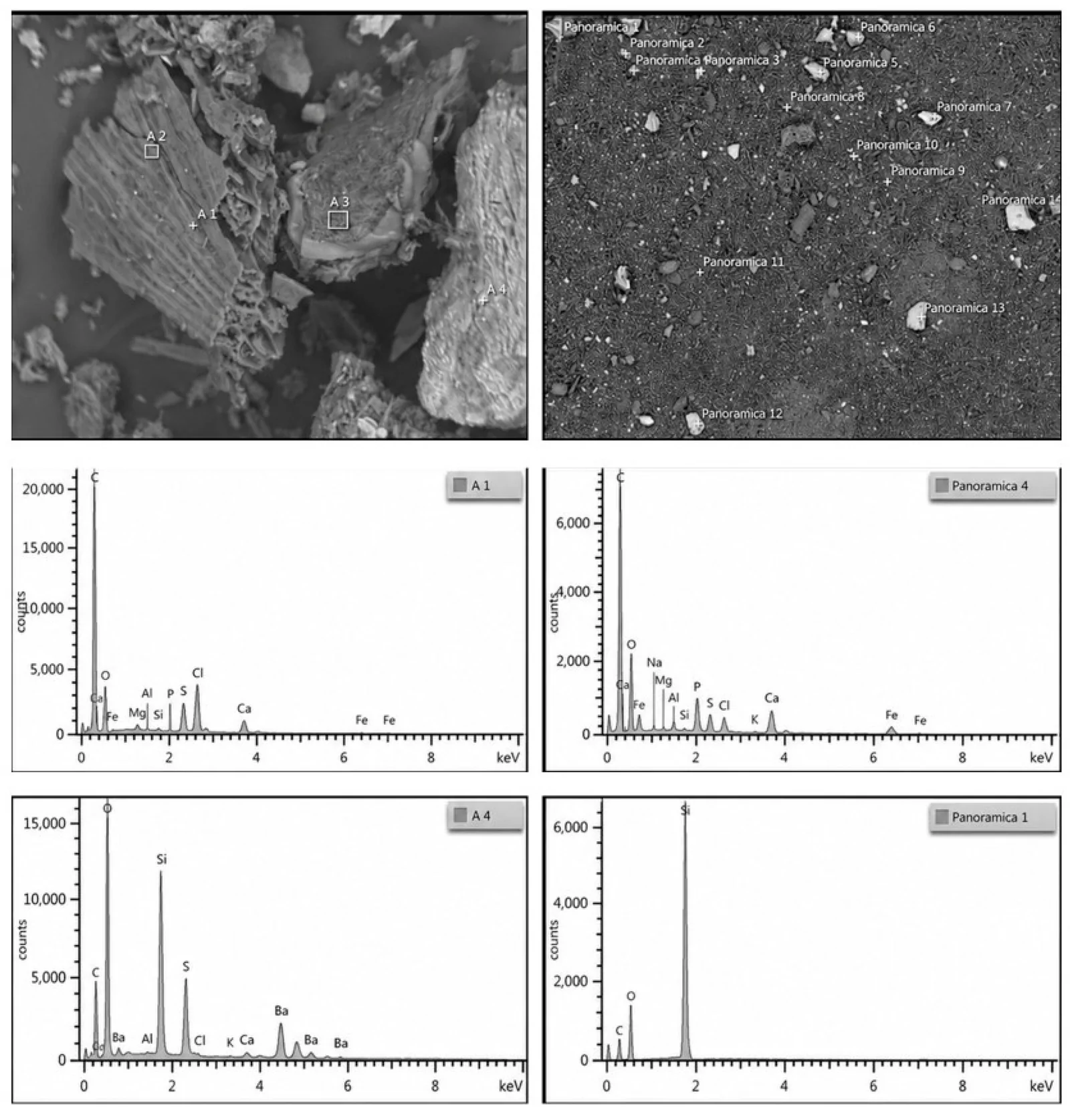
SEM details and EDS spectra related to BC400S and BC400SU.

Interestingly, energy-dispersive X-ray spectroscopy (EDS) analysis demonstrated a homogeneous distribution of sulfur across both the carbonaceous matrix and the mineral phases in the sulfonated samples (see elemental mapping reported in Figure A6). However, after catalytic use, sulfur was retained only on the carbonaceous support, whereas the sulfur initially associated with the silica-rich mineral fraction was no longer detected, indicating that it had been leached during the catalytic process (Figure A7).

### 2.4. Reactivity of the Sulphonated Biochars in the Direct Esterification

BC400S and BC550S were evaluated as solid acid catalysts for the direct esterification of free fatty acids (FFAs) recovered from sewage sludge, as described in Section 2.1. Prior to the reaction, the recovered grease was subjected to vacuum distillation (190 °C, 2 mmHg) to leave heavy compounds and minerals, which could affect the catalyst’s activity [35]. After this operation, the FFA content exceeded 99%. The use of mild reaction conditions, with a maximum temperature of 80 °C, ensured complete selectivity toward methyl esters (100%), with the product yield corresponding to the FFA conversion. Figure 3a shows the esterification kinetics obtained with BC400S and BC550S, compared with those of the blank experiment (performed without catalyst) and two benchmark acid catalysts, namely the conventional heterogeneous resin Amberlyst 15 [36] and homogeneous sulfuric acid (H_2_SO_4_). To enable a meaningful comparison, all catalytic systems were employed at the same acid site loading, corresponding to 0.05 meq of acid sites per gram of grease. As expected, homogeneous H_2_SO_4_ exhibited the highest catalytic activity owing to the complete accessibility of its acid sites. Among the heterogeneous catalysts, the activity followed the order BC400S ≥ BC550S > Amberlyst 15, demonstrating the superior catalytic performance of the sulfonated biochar-based catalysts under the investigated reaction conditions.

It is worth noting that the pristine biochars, BC400 and BC550, produced only negligible amounts of fatty acid methyl esters compared with their corresponding sulfonated counterparts (FFA conversion in the range of 22–29%). This finding demonstrates that the intrinsic surface acidic functionalities of the untreated biochars were not sufficiently strong to effectively catalyze the esterification reaction under the mild operating conditions adopted (75 °C). Therefore, the introduction of sulfonic acid (–SO_3_H) groups through sulfonation was essential to impart the strong Brønsted acidity required for efficient direct esterification.

**Figure 4:**
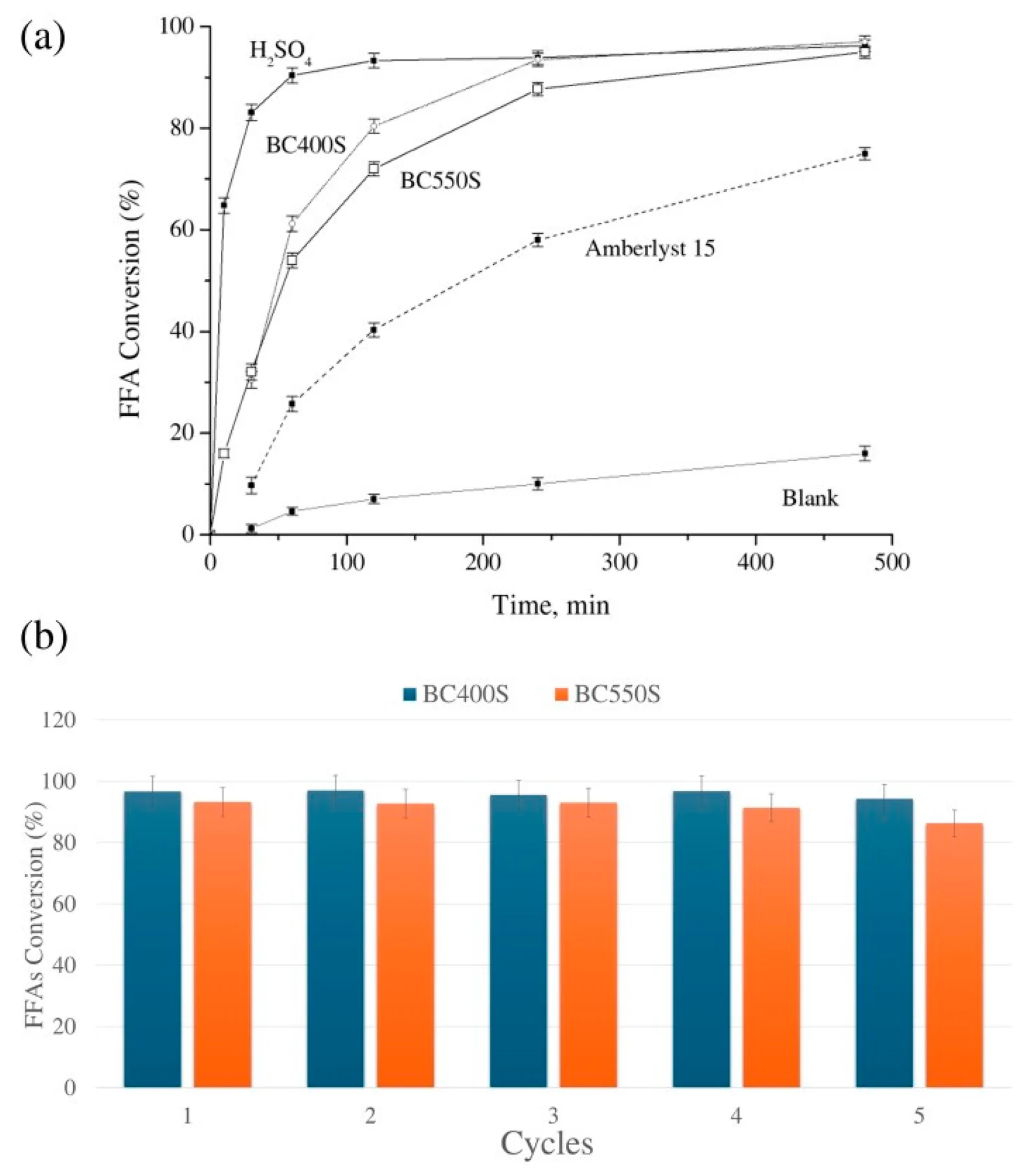
(**a**) Kinetics of direct esterification FFAs:MeOH 1:10 molar, 75 °C, catalyst loads: 0.05 meq of acid sites per gram of grease. (**b**) Recycling of catalysts (FFAs:MeOH 1:10 molar, 80 °C, 5 h, catalyst load: 3 wt.% of grease).

Another noteworthy aspect is the robustness and stability of the sulfonated biochar (BCS) catalysts. For this specific test, with the aim of reducing the reaction time (5 h) while maintaining a reasonable conversion, the temperature was increased to 80 °C, using a catalyst loading of 3 wt.% relative to the grease. Both catalysts could be readily reused for five consecutive reaction cycles without significant loss of catalytic activity. Catalyst recovery was achieved simply by centrifugation to separate the reacted organic phase. The resulting wet solid was directly resuspended in a fresh methanol/FFA mixture and reused for a subsequent 5 h esterification cycle without any intermediate regeneration treatment. As shown in Figure 4b, BC400S exhibited slightly higher catalytic activity and stability than BC550S throughout the recycling tests. At the end of the fifth reaction cycle, the recovered catalysts were washed with methanol, dried, and subsequently characterized by scanning electron microscopy (SEM). As previously observed (Figure A6), the pristine sulfonated biochars consisted of a heterogeneous carbonaceous matrix containing dispersed silica-rich mineral particles, with sulfur homogeneously distributed over both the carbonaceous and mineral phases. After reuse, the catalysts still retained a high sulfur content overall. However, localized energy-dispersive X-ray spectroscopy (EDS) analyses (Figure A7) revealed that a significant fraction of the sulfur initially associated with the mineral particles had been leached during the catalytic cycles, whereas the sulfur grafted onto the carbonaceous matrix remained largely preserved. The retention of these carbon-bound sulfonic acid groups was sufficient to maintain the initial catalytic performance over repeated uses.

## 3. Discussion

**Figure 5:**
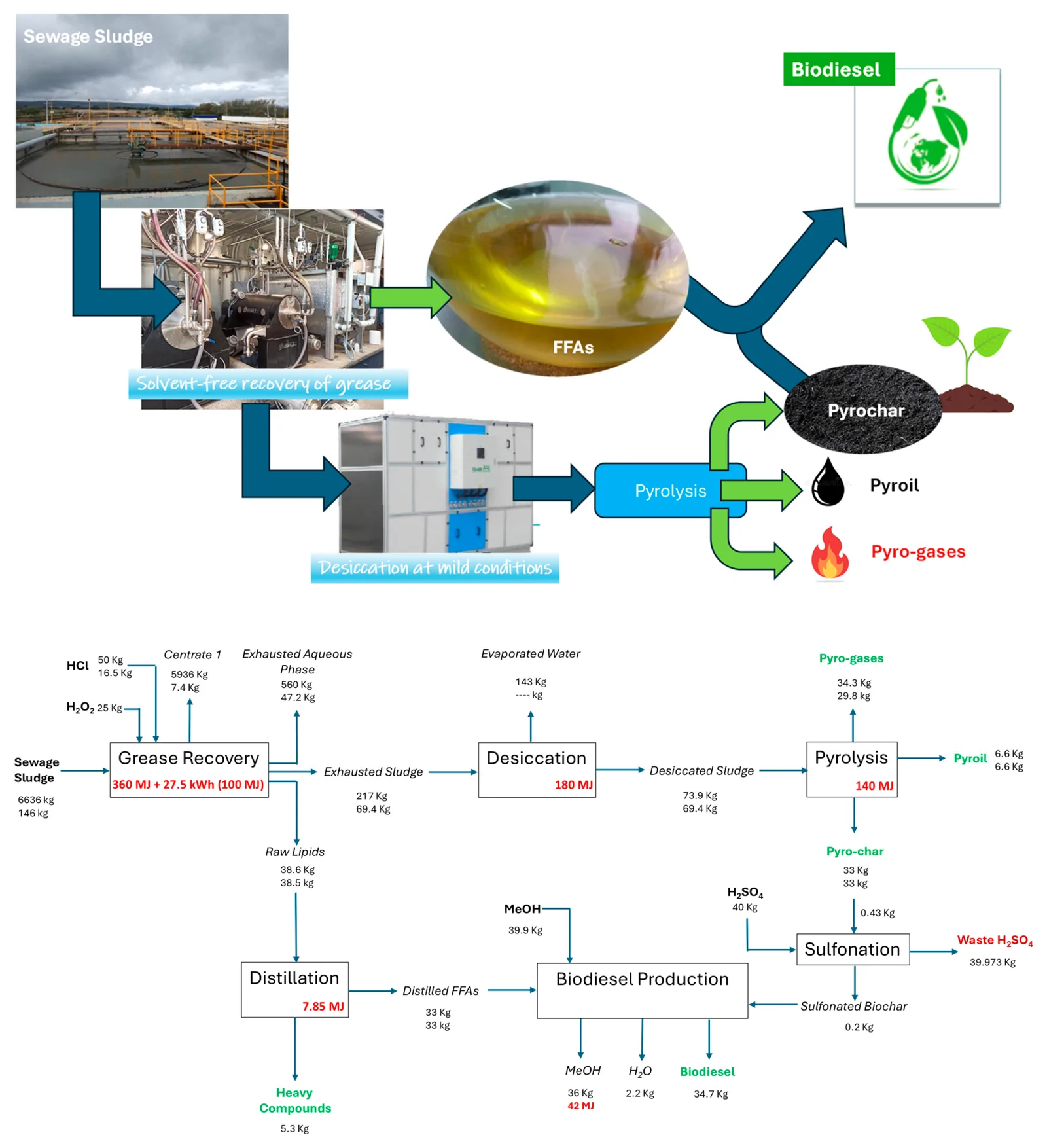
Integrated process for the complete valorization of sewage sludge. Solvent-free grease recovery produces a free fatty acid (FFA)-rich stream for biodiesel production and a degreased sludge, which is subsequently dried and converted by pyrolysis into pyro-char, bio-oil, and pyro-gas, enabling both material and energy recovery within a circular economy framework (in correspondence with sewage sludge and each reagent/intermediate/product, the relevant wet and dry masses were reported).

Figure 5 reports a schematic representation of the integrated valorization pathway developed in this work for urban sewage sludge, with a specification of currents and relevant masses.

Sewage sludge is first subjected to a solvent-free grease recovery process, yielding a free fatty acid (FFA)-rich fraction, which was distilled under low pressure (190 °C, 2 mm Hg) to produce pure FFAs (>99%), and a degreased sludge. The recovered FFAs are subsequently converted into biodiesel through catalytic esterification. Meanwhile, the residual sludge is desiccated under mild conditions and then subjected to pyrolysis, producing three value-added products: pyro-char, which can be used as a carbon-rich material for environmental and catalytic applications for the valorization of the separated grease into biodiesel after its sulfonation; pyrolysis oil (bio-oil); and pyro-gas (syngas), which can be recovered as renewable chemicals and energy sources. This integrated approach maximizes the recovery of both matter and energy from sewage sludge, supporting a circular economy strategy and minimizing waste generation. In detail, according to the presented results, the recovery of matter was optimized if pyrolysis was run at 400 °C, increasing the recovery of biochar and pyro-oil. In addition, the biochar obtained at 400 °C produced the best precursor for the generation of the most active sulfonated system (BC400S), to be used in catalysis for the direct esterification of FFAs.

The results demonstrate that integrating solvent-free grease recovery with pyrolysis provides an effective strategy to maximize carbon valorization from sewage sludge, while preserving as much as possible the initial organic compounds. Unlike conventional sludge management, where the residual biomass is mainly directed to anaerobic digestion or combustion, the proposed cascade approach enables the simultaneous recovery of valuable products while retaining part of the carbon in a stable solid phase and grease, which can be converted into biodiesel, which can substitute for fossil-derived fuel. It is worth noting that sewage sludge represents the only carbon source in the process. Apart from methanol, whose unreacted fraction can, in any case, be recycled, sewage sludge is used to recover pyro-oil, pyro-char, heavy organic compounds, and biodiesel. This hierarchy of valorization is consistent with the principles of the revised Urban Wastewater Treatment Directive [37], which encourages the recovery of valuable resources from wastewater and sewage sludge while reducing the environmental footprint of wastewater treatment plants. Centrate 1, obtained from the first dewatering step of the sedimented sludge (5936 kg from an initial 6636 kg), the exhausted aqueous phase (560 kg), the water evaporated during the drying process (146 kg), and the water generated during the direct esterification reaction (2.2 kg), could potentially be directly recirculated to the influent of the WWTP. The only significant waste stream apparently generated by the process arises from the sulfonation of the biochar and consists of unreacted sulfuric acid. However, this acid could potentially be recovered and recycled for the production of new sulfonated catalysts. Further dedicated investigations are required to better assess the effective environmental impact and management of this side stream.

From a process perspective, the solvent-free extraction recovered a substantial fraction of the free fatty acids for biodiesel production, whereas the remaining carbon-rich residue was successfully converted by pyrolysis into pyro-char, bio-oil, and pyro-gas. The product distribution was strongly influenced by the operating temperature. Lower temperatures (400 °C) favored the formation of bio-oil rich in residual free fatty acids, whereas increasing the temperature progressively shifted the carbon and energy balance toward the gaseous fraction. At 750 °C, the pyro-char composition approached the ash content of the original feedstock, indicating extensive decomposition of the organic matrix. These observations agree with previous reports showing that pyrolysis temperature is the primary factor controlling the distribution of solid, liquid, and gaseous products.

From an energy perspective, Figure 5 reports the estimated specific energy requirements for each unit operation, referring to a single batch cycle of sewage sludge treatment designed to process 700 kg of centrifuged sludge. The solvent-free grease recovery step requires approximately 360 MJ of thermal energy and 27.5 kWh (100 MJ) of electrical energy for the associated equipment [7]. The drying step requires approximately 50 kWh (180 MJ) [8], while the energy demand for pyrolysis was assumed to be 2 MJ kg^−1^ of dried sludge, corresponding to approximately 140 MJ [38].

In addition, the energy required for the distillation of FFAs (0.237 MJ kg^−1^, corresponding to 7.85 MJ [39]) and for the evaporation and recovery of unreacted methanol (42 MJ) [40] were considered. Based on these assumptions, the overall energy consumption associated with the proposed process network was estimated to be approximately 830 MJ.

It is worth noting that this preliminary assessment does not account for potential energy integration and heat recovery between the different process units. Therefore, the calculation represents a conservative, worst-case scenario in which the entire energy demand must be externally supplied. Even under these pessimistic assumptions, the energy associated with the BC400 biochar would be sufficient to cover the overall energy requirement. Indeed, using the Dulong equation [41] to estimate the higher heating value (HHV) of BC400, a value of 27.88 MJ kg^−1^ was obtained, corresponding to an overall energy content of approximately 920 MJ.

These preliminary results suggest that the energy contained in BC400 could potentially sustain the overall energy demand of the proposed process. Consequently, the most valuable products, namely biodiesel, heavy organic compounds, and pyro-oil, could potentially be recovered without the need for an external energy supply, assuming efficient utilization of the energy contained in the biochar.

Beyond energy recovery, the production of pyro-char represents one of the most valuable outcomes of the proposed process. In fact, only a very limited amount needed to be converted in the catalyst through sulfonation (0.43 kg of 33 kg, namely 1.3%), whereas it could be mostly used as an in situ energy feedstock for feeding the energetic need of the entire network, or alternatively, in agriculture, or in cement industries, completing the sustainable turning of a waste into resources. The pyrolytic treatment is, in fact, functional to the thermic degradation of organic pollutants adsorbed onto sewage sludge, a problem often ignored, but that in the future could hamper the direct use in agriculture of conventionally obtained sewage sludge. Even if it was not specifically investigated in the present work, it was already demonstrated that the pyrolysis of sewage sludge run at 400–550 °C effectively removes most (over 95%) of emerging and persistent pollutants [42,43]. In addition, its successful functionalization through sulfonation enabled its reuse as a heterogeneous acid catalyst for biodiesel production. The catalytic tests demonstrated that BC400S exhibited higher activity than BC550S and outperformed the commercial Amberlyst 15 resin under identical acid site loading.

Table 5 reports an overview of several sulfonated biochars reported in the literature with which the results reported in the present work were compared.

According to these data, BC400S and BC550S’ activities are in line with the results previously reported for analogous systems.

If compared with conventional sulfuric acid, it could allow recovery and reuse at least five times of a species that, if not anchored, would be lost in a single use, since H_2_SO_4_ is hardly recoverable from the reaction mixture of direct esterification. Moreover, both catalysts maintained their activity over five consecutive reaction cycles, confirming that the sulfonic acid groups anchored to the carbonaceous matrix remained stable despite partial sulfur leaching from the mineral fraction. This result highlights the importance of the carbonaceous framework as an effective catalytic support. This aspect addresses the sustainability and atom economy hierarchy, two of the main pillars of Green Chemistry [50].

The proposed integrated process therefore combines material recovery, energy production, and catalyst synthesis within a single wastewater treatment scheme. Such an approach contributes not only to resource circularity but also to the decarbonization objectives established by the revised European legislation. By recovering free fatty acids for biodiesel production, converting the residual biomass into renewable energy carriers, and generating reusable carbon-based catalysts, the process minimizes waste generation while maximizing the value extracted from sewage sludge. This integrated carbon management strategy may represent a viable pathway toward energy-neutral and resource-positive wastewater treatment plants.

## 4. Materials and Methods

### 4.1. Materials and Precursors

#### 4.1.1. Reagents

Different laboratory-grade reagents were used, such as hexane, ethanol, methanol, barium chloride (BaCl_2_), potassium bromide (KBr), sodium hydroxide (NaOH), potassium hydroxide (KOH), HCl (33 wt.%), H_2_O_2_ (35 wt.%), Sulfuric acid (H_2_SO_4_), and phenolphthalein, all of which were purchased as reagent grades from Aldrich-Merk, St. Louis, MO, USA.

#### 4.1.2. Sewage Sludge and Solvent-Free Grease Recovery

The sludge was collected during the primary treatment stage of a wastewater treatment plant (WWTP) that processes wastewater generated by daily urban activities in the city of Lecce (120,000 People Equivalent). Specifically, the sludge was treated as reported by di Bitonto et al. in recent works [7,8], to recover the grease component. Sewage sludge was dewatered using a horizontal centrifuge and collected in a reactor, where centrifuged sludge were mixed with HCl (33 wt.%) and H_2_O_2_ (35 wt.%), heated to 80 °C and centrifuged again with a horizontal decanter producing a liquid phase and an exhausted solid sludge (TS 32%), which was desiccated by a SCHINCCI apparatus that allowed the obtainment of a final sludge with a residual humidity <6%. The liquid phase was immediately processed by a vertical separator, which allow to recover the raw grease (C_10_–C_22_ content > 90%, yield recovery 72 wt.%). The desiccated solids were chemically characterized in terms of residual lipids, structural carbohydrates (easily hydrolysable sugars and cellulose), proteins, and ashes according to well define protocol of analysis reported in di Bitonto et al. 2024 [7].

### 4.2. Pyrolysis (Pyro-Char)

Pyrolysis experiments were conducted in a tubular furnace under a continuous nitrogen (N_2_) flow to maintain an inert atmosphere. The system was operated using 10 g of lipid-free primary sewage sludge residue with a heating rate of 10 °C/min up to target temperatures of 400 °C, 550 °C, or 750 °C, which were maintained for 2 h. After cooling under N_2_, the yields of pyro-char and bio-oil were quantified, whereas the gaseous phase was determined by difference.

### 4.3. Catalyst Functionalization

The carbonaceous matrices (pyro-chars) were sulfonated using concentrated H_2_SO_4_ at 175 °C to graft sulfonic groups onto the surface. In a typical procedure, 3 g of carbonaceous material was reacted with 150 mL of concentrated H_2_SO_4_ (98%). The mixture was heated to 175 °C and maintained under constant stirring for 24 h. After the reaction, the system was allowed to cool to room temperature and subsequently diluted to a total volume of 500 mL using deionized water. The resulting solid was recovered by filtration and intensively washed with deionized water. The washing process continued until the filtrate reached the absence of residual sulfate ions in the filtrate was confirmed using the barium chloride (BaCl_2_) test (no precipitation of BaSO_4_ observed). Finally, the sulfonated catalysts were dried at 80 °C (1.4 g, yield 46.7%) and subsequently employed in esterification reactions.

### 4.4. Catalysis of the Esterification

The synthesized catalysts were subsequently tested in esterification reactions with methanol for producing biodiesel (fatty acid methyl esters), directly testing the grease recovered through the solvent-free procedure from sludge described above, after a preliminary distillation under vacuum (190 °C, 2 mm Hg). The initial comparison among different catalysts was run working at 75 °C and using an acid load of 0.05 meq·g^−1^ grease, which corresponded to approximately 3.74 wt.% BC400S, 5.40 wt.% BC550S, 0.5 wt.% H_2_SO_4_, and 2.5 wt.% Amberlyst 15 (the concentration of acid sites reported in Table 4 was considered). Recycling tests were carried out at 80 °C for 5 h, using a fatty acid/methanol molar ratio of 1:10 and a catalyst loading of 3 wt.% relative to oil mass. Upon completion, the mixture was cooled to room temperature to observe potential phase separation. Conversion yields were determined via Gas Chromatography (GC-FID) and, for esterification, also validated by acid-base titration using a 0.10 M KOH solution. Both experiments were repeated two times. Figure 4 reports the average values with the interval of variability assessed.

### 4.5. Analytical Techniques

Analytical validation was provided by Fourier Transform Infrared Spectroscopy (FTIR) to monitor functional group changes, X-Ray Diffraction (XRD) for crystalline phase identification, and Gas Chromatography (GC-FID) to determine the final biodiesel conversion yields.

Elemental analysis of ashes and lipid-free residues was conducted using Inductively Coupled Plasma Optical Emission Spectroscopy (ICP-OES) with a Thermo Scientific iCAP 7000 series (Thermo Fisher Scientific, Bremen, Germany) to determine the concentration of major and trace elements. Samples were digested in concentrated HNO_3_ and then diluted to appropriate concentrations before analysis. Duplicate measurements were performed to ensure analytical reproducibility, with relative standard deviations below 5% for all reported elements.

CHNS analyses were determined using an NC Instrument model 4024 (NC Technologies, Bussero, Italy).

The specific surface area and pore analysis were determined using N_2_-physisorption at liquid nitrogen temperature on a TriStar II instrument (Micromeritics, Norcross, GA, USA). The samples were degassed for 8 h at a temperature of 350 °C (with a heating rate of 5 °C⋅min^−1^) on a SmartVac Prep device (Micromeritics, USA). Both adsorption and desorption isotherms were measured.

The acid properties of the samples were analyzed using temperature-programmed desorption of ammonia (TPD-NH_3_) on an AutoChem II 2920 (Micromeritics, USA) [51]. Before each measurement, the sample was pretreated in a helium stream (25 cm^3^⋅min^−1^) at 180 °C. For TPD-NH_3_, the adsorption of the respective gas (5% NH_3_/He; 25 cm^3^⋅min^−1^) was taken at 35 °C and 70 °C, respectively, followed by removal of physisorbed molecules in a helium stream (25 cm^3^⋅min^−1^) and finally the desorption analysis in a He stream (25 cm^3^⋅min^−1^) with linear heating at 10 °C⋅min^−1^.

X-ray diffractograms were obtained in the 5–85 °2θ range using a Malvern-Panalytical Empyrean diffractometer equipped with a Medipix3 PIXel 1D detector (Malvern Panalytical B.V., Almelo, The Netherlands). The instrument was operated at 45 kV and 40 mA, employing the Bragg–Brentano configuration and CuKα1 radiation (λ = 1.5406 Å). Samples were analyzed using the flat sample workstation with a sample size and passage time of 0.026 °2θ and 96.36 s, respectively. The incident beam optics included a 0.04 rad Soller slit, a 10 mm fixed mask, a fixed 1/2° divergence slit, and a fixed 2° antiscatter slit. On the other hand, in the diffracted beam optics, a 0.04 rad Soller slit and a 7.5 mm anti-dispersion slit were used. Phase identification was performed using HighScore Plus software Version 4.9. Functional group analysis of catalyst precursors and functionalized materials was conducted using a Nicolet iS 10 FTIR (Thermo Fisher Scientific, Madison, WI, USA) in the 4000–400 cm^−1^ range. Spectra were recorded with a resolution of 4 cm^−1^ and 32 scans per spectrum. For the carbonized materials and ashes, KBr was used as a medium in the preparation of pellets, which considerably improved the spectra of the synthesized materials.

Morphology and microanalysis were obtained by SEM-EDS using a ZEISS EVO 50XVP microscope (ZEISS, Oberkochen, Germany) equipped with an Oxford-Link detector (Link Gulf LLC, Oxford, UK). Samples were mounted on carbon disks and coated with graphite; selected samples were analyzed uncoated to avoid coating-related artifacts. Measurements were performed at 20 kV and 500 pA. Spectra were acquired with a 100× objective, 10 s exposure time, two accumulations, and a 532 nm laser (12.5 mW, 50% power). No laser-induced damage was observed. Spectral acquisition was performed in the 100–1200 cm^−1^ range, and baseline correction was applied prior to analysis. Esterification and transesterification conversion yields were quantified by Gas Chromatography with Flame Ionization Detection (GC-FID) analysis. An aliquot of 1 mL of the reaction system was diluted with 1 mL of a methyl heptadecanoate methanolic solution (2 mg/mL). An Agilent 8890 GC-FID was equipped with an HP-5MS capillary column (30 m; Ø 0.32 mm; 0.25 μm, Agilent Technologies, Santa Clara, CA, USA). Injections were performed in splitless mode, with an injector temperature equal to 250 °C, and helium was used as a carrier gas, with a flow of 2.8 mL/min. The temperature of the internal oven was initially set to 60 °C and held for 4 min; then, the temperature was increased to 200 °C (rate of increase 10 °C/min), held for 1 min, and the final temperature of 250 °C (rate of increase 20 °C/min), held for 3 min. The temperature of the detector (FID) was set to 340 °C. The quantification of fatty acid methyl esters (FAME) was performed according to Equation (1):

(1) $$\mathrm{FAME}\;(\mathrm{wt}.\%)\;=\;\frac{\sum A_{i}}{A_{S}}\cdot \frac{w_{s}}{w_{TS}}\cdot \;100$$

where A_i_ is the area of the i-th fatty acid methyl ester, A_S_ and *w_S_* are the area and the amount (g) of the internal standard methyl heptadecanoate, respectively, and *w_TS_* is the amount of the starting sample (g).

Qualitative determinations were recorded using a GC-MS Shimadzu with a configuration reported for GC-FID.

## 5. Conclusions

This work demonstrated the technical feasibility of an integrated biorefinery approach for the comprehensive valorization of urban sewage sludge, combining solvent-free grease recovery, biodiesel production, and thermochemical conversion of the residual biomass. The solvent-free extraction process successfully recovered approximately 72 wt.% of the grease contained in sewage sludge, producing a free fatty acid-rich stream suitable for biodiesel production while avoiding the use of organic solvents.

The residual degreased sludge was effectively upgraded through low-temperature drying followed by pyrolysis. Pyrolysis temperature strongly influenced product distribution: operation at 400 °C maximized the production of bio-oil rich in residual free fatty acids, whereas increasing the temperature promoted gas formation and yielded a mineral-rich pyro-char. The resulting pyro-char was successfully functionalized by sulfonation to produce reusable solid acid catalysts.

Among the investigated materials, BC400S exhibited the highest sulfur loading and the best catalytic performance in the esterification of recovered free fatty acids, outperforming the commercial Amberlyst 15 catalyst under identical acid site loading. Furthermore, the catalyst maintained its activity over five consecutive reaction cycles without regeneration, demonstrating excellent stability despite the partial leaching of sulfur species associated with the mineral fraction. EDS analyses confirmed that the catalytically active sulfonic groups remained preferentially anchored to the carbonaceous matrix, preserving the catalyst performance.

Overall, the proposed process represents an efficient carbon management strategy for wastewater treatment plants, maximizing the recovery of both materials and energy while minimizing waste generation. Beyond biodiesel production, the conversion of the residual biomass into biochar, bio-oil, and syngas significantly enhances the overall valorization of sewage sludge and contributes to carbon retention within stable solid products. The integration of resource recovery with catalyst production establishes a closed-loop approach that is fully consistent with circular economy principles and supports the transition of wastewater treatment plants toward resource-positive and energy-neutral facilities, as envisaged by the revised European Urban Wastewater Treatment Directive [37].

Future work should focus on pilot-scale continuous operation, comprehensive techno-economic and life-cycle assessments, and the long-term evaluation of catalyst durability and contaminant fate to further assess the industrial applicability of the proposed integrated process.

The proposed integrated platform transforms sewage sludge from an environmental liability into a renewable carbon resource, enabling the simultaneous production of biofuels, reusable catalysts, and renewable energy carriers while supporting the transition toward circular and energy-neutral wastewater treatment plants.

## Figures and Tables

**Table 1:** Chemical composition of the centrifuged sewage sludge after the chemical extraction of the lipid component.

Total Solid Composition	(g/Kg)
Total lipids	117
Easy hydrolysable sugars (EHS)	
Galactosamine	2
Arabinose	2
Glucosamine	2
Galactose	3
Glucose	20
Mannose + xylose	27
Total	56
Cellulose	
Glucose	161
Mannose + xylose	13
Total	174
Protein	197
Lignin-Humic compounds	234
Ash	185
Total	962

**Table 2:** Elemental composition (mg/kg) of primary sewage sludge lipid-free residue determined by ICP-OES.

Element	Content (mg/kg)
Al	4629.65
B	73.21
Ba	120.92
Ca	20,578.24
Cr	409.47
Cu	235.28
Fe	5757.64
K	900.21
Mg	2988.75
Mn	63.66
Mo	18.26
Na	1316.73
Ni	159.93
Pb	95.07
Si	1697.31
V	21.44
Zn	478.44

**Table 3:** Distribution of pyro-chars, pyro-oils, and gaseous phases during pyrolysis at 400, 550, and 750 °C.

	T400	T550	T750
Pyro-char	47.5 ± 2.5%	34.7 ± 3.2%	31.6 ± 2.5%
Pyro-oil	9.5 ± 2.1%	1.0 ± 0.5%	0
Gases	43.0 ± 2.2%	64.3 ± 3.5%	68.4 ± 2.5%

**Table 4:** CHNS composition and concentration of acid sites (CAS) determined through TPD-NH_3_ of biochars and the relevant sulphonated compounds.

	BC400	BC550	BC400S	BC550S
C	40.4 ± 1.8	38.42 ± 0.7	39.4 ± 0.5	44.6 ± 0.3
H	2.8 ± 0.3	1.75 ± 0.02	2.3 ± 0.2	1.5 ± 0.1
N	3.4 ± 0.1	2.8 ± 0.1	2.9 ± 0.1	3.3 ± 0.1
S	-	-	2.2 ± 0.1	1.6 ± 0.1
CAS (μmol·g^−1^)	456	533	1336	926

**Table 5:** Literature overview on production, derivatization and reactivity of sulfonated biochars from different biomasses.

Entry	Biomass	Pyrolysis	Sulfonation	Organic Acids	Reactive Conditions	Yield	Ref.
		T (°C)/t (h)	Reagent/T (°C)/t (h)				
1	Corncob	300/3	H_2_SO_4_/90/2	Oleic Acid	12:1/65/4/10	98.5	[44]
2	Potato peel	500/2	H_2_SO_4_/180/8	Oleic Acid	12:1/80/2.5/5	97.2	[45]
3	Rice husk	700/3	ClSO_3_H	Oleic Acid	16:1/75/5/9.9	98.1	[46]
4	Empty fruit bench	500/-	H_2_SO_4_/90/1	Palm fatty acid distillate	15:1/75/15/5	95	[47]
5	Coconut shell biochar	-	H_2_SO_4_/150/12	Palm fatty acid distillate	8:1/60/2/10	75	[48]
6	Peanut Shell	-	H_2_SO_4_/85/3	Oleic Acid	10:1/85/6/7	98	[49]
7	Sewage sludge	400/2	H_2_SO_4_/175/24	Sewage FFAs	10:1/80/5/3	96.7	This work
8	Sewage sludge	550/2	H_2_SO_4_/175/24	Sewage FFAs	10:1/80/5/3	93.2	This work
9	Sewage sludge	400/2	H_2_SO_4_/175/24	Sewage FFAs	10:1/75/8/3.4	97	This work

## Data Availability

The raw data supporting the conclusions of this article will be made available by the authors on request.
